# Supplementary material for: Positive Patient Postoperative Outcomes with Pharmacotherapy: A Narrative Review including Perioperative-Specialty Pharmacist Interviews
Source: J Clin Med. 2022 Sep 24;11(19):5628. doi: 10.3390/jcm11195628 (PMC9572852; doi:10.3390/jcm11195628)
Supplement: Supplementary file 1 [file jcm-11-05628-s001.zip › jcm-1907200 supplymentary.pdf]

Supplemental Material S1.

Practice Examples Illustrating Collaborative Care and Drug Stewardship for Improved Medication Management and Patient Outcomes

The following pharmacists currently work or have worked in a collaborative care relationship with patients and the perioperative team with varying degrees of formalization. Each was interviewed via Zoom, and an edited transcript was generated to illustrate the development of advanced practice or clinical pharmacy within major surgical specialty areas. The terms, "advanced practice" and "clinical" pharmacist are used interchangeably and denote a practice focused on direct patient care. A description of their practices follows that serves to identify drivers and strategies for making improvements in medication management for perioperative patients through drug stewardship activities and informal or formal collaborative pharmacotherapy practice enhancements along the surgical care continuum. In addition to collaborative practice and drug stewardship, many of these pharmacists engaged in joint scholarly activities with the perioperative team(s) to which they were attached. Supplemental Table S1 identifies the perioperative clinical pharmacists and their specialty area that were interviewed. For template examples and resources for implementing collaborative practice, see the websites of the National Association of State Pharmacy Associations (https://naspa.us/resource/swp/) and the Centers for Disease Control and Prevention (https://www.cdc.gov/dhdsp/pubs/docs/CPA-Translation-Guide.pdf) for more information.

**Specialty area**	**Perioperative clinical pharmacist**	**Organization**
General perioperative	Sara J. Hyland, PharmD, BCCCP	Grant Medical Center / OhioHealth
Bariatrics	Alyssa Pollock, PharmD, BCPS	Creighton University / CHI-Health
Cardiothoracic	William E. Dager, PharmD, BCPS	University of California Davis
Colorectal	Jenna K. Lovely, PharmD, BCPS	Mayo Clinic - Rochester
Gynecological oncology	Siu-Fun Wong, PharmD	University of California Irvine Health
Orthopedics	Dustin Carneal, PharmD	Crystal Clinic Orthopedic Hospital
Pediatrics	Heather Monk Bodenstab, PharmD	Children's Hospital of Philadelphia
Solid organ transplantation	Tracy M. Sparkes, PharmD, BCTXP	University of Maryland Medical Center
Vascular	Ryan Cassity, PharmD	Mayo Clinic - Rochester

Supplemental Table S1: Perioperative Advanced Practice or Clinical Pharmacists Interviewed. (Note: BCCP = board- certified critical care pharmacist; BCPS = board-certified pharmacotherapy specialist; BCTXP = board-certified solid organ transplantation pharmacist)

General perioperative - Sara Jordan Hyland, PharmD, BCCCP, OhioHealth/Grant Medical Center, Columbus, OH

Collaborative practice at Grant started in 2012 when I was a pharmacy practice resident. I wanted to do an elective rotation during my residency and became interested in the opportunities in perioperative care. At that time, pharmacy operations in perioperative areas were mostly centralized with no clinical pharmacy presence. An anesthesiologist, who had always supported collaborative efforts and welcomed having increased pharmacy support, served as my rotation preceptor. In the 4-week rotation, I shadowed the anesthesiologist throughout the day in theater and on rounds. Soon, it became apparent that a clinical pharmacist was needed and wanted in our perioperative area. Multiple opportunities for process improvement and clinical intervention existed that even a pharmacy resident could make. For example, a

surgeon, in the middle of the case, wanted to give cefazolin every two hours to a patient who was a seizure risk and renally impaired. I was able to assist the anesthesiologist to intervene and make a case for patient safety, noting that the patient was adequately covered based on the procedure type and blood loss. By the end of the rotation, the OR began advocating for improved medication processes and assignment of a "go-to" clinical pharmacy resource. A year later, I collaborated with the pharmacy director and anesthesiologist leadership to make the case for a dedicated perioperative pharmacist resource.

Stewardship started with perioperative antibiotics which continues to be a huge focus of our service improvement that culminated in several American Academy of Orthopedic Surgeons publications [111,157]. Compliance with CMS SCIP (surgical care improvement program) guidelines for antibiotic selection and timing was suboptimal, and reimbursement dollars were lost. All preoperative antibiotics are now prospectively reviewed and optimized for inpatient and outpatient surgeries, penicillin allergies are assessed and de-labeled, and first line antibiotics are used with minimization of broad-spectrum antibiotics. By comprehensively optimizing perioperative antibiotics and associating that practice with a lower surgical site infection rate, the service grew to three pharmacist FTEs and two pharmacy technician FTEs. Pharmacy collaboration helped to improve patient outcomes with a huge cost savings for the hospital. Another stewardship area has been high cost and relatively low value medications, for example, liposomal bupivacaine (LB) in orthopedics. A collaborative prospective randomized controlled trial with orthopedic surgeons was performed that demonstrated that LB did not contribute positively to any patient-relevant outcome. Results were published in the Journal of Arthroplasty [74]. The stewardship effort for LB continues across multiple certain service lines. Joint publications have been an excellent vehicle for documenting and sustaining collaborative activities and efforts.

Another high-risk, high-cost medication use primed for stewardship was blood coagulation factor products. A completely new treatment algorithm for refractory bleeding in cardiothoracic surgery, addressing whole patient management, applying thromboelastography and other supportive measures, as opposed to focusing solely on a cost savings or medication stewardship. Publication of these results is pending later this year. In addition, the direct neuromuscular blockade reversal agent, sugammadex, has revolutionized modern anesthetic management, but indiscriminate use doesn't always add value for the patient. Determining who actually benefits and collaborating with anesthesiologists to make data driven decisions is a current area of focus.

Opioid stewardship is one of the latest stewardship efforts, in the wake of the opiate epidemic, to optimize analgesic strategies and opioid exposure throughout the perioperative process. When perioperative clinical pharmacists began working with the orthopedic service line at my institution, all patients received oxycodone and IV PCA as the sole postoperative analgesic modalities. Best practices for managing patients with chronic pain or opioid use disorders prior to admission were derived, and preemptive analgesic protocols in the surgery center were leveraged. Clinical pharmacy collaborated longitudinally with anesthesiology to develop an enhanced recovery analgesic anesthetic pathway to optimize regional anesthesia and worked with the surgeons drastically reduce opioid discharge prescription quantities as well as facilitate earlier discharge [112].

Outcomes measurement is very important and requires team and quality administrative support that varies widely by surgical service line. A more robust data collection machine is needed to support enhanced recovery efforts. Colorectal, for example, has dedicated quality improvement meetings and personnel to track and trend recovery of bowel function and length of stay. In addition, many enhanced recovery efforts have been pharmacist-driven collaboratives with data. In orthopedics, for example, when the preemptive analgesia protocol and standardized anesthetic enhanced recovery analgesics pathway was activated, pharmacy collected data, derived an evidence-based protocol, and benchmarked the percentage of patients

ambulating with physical therapy successfully on day of surgery to reach physical therapy goals and facilitate discharge.

A pre-admission pharmacist is in the planning stage based on strong relationships with pre-admission physicians and nurses, now an informal collaborative relationship without a dedicated FTE. For example, obtaining documented allergy information from pre-admission testing has enabled that the pharmacist to optimize antibiotics before surgery and address smoking cessation with a nicotine replacement program. Case series data are collected on preventable adverse events that the pharmacist could have identified to build a case for additional dedicated resources to improve perioperative medication management. The PACU is covered by perioperative pharmacists, as well as preoperative and intraoperative phases of care. For all of these different procedural areas, it's advantageous to have one dedicated pharmacist who was involved in decision-making in preop and how that affects PACU.

Collaborative practice agreements (CPAs) where the clinical pharmacist is accountable and responsible for medication management in perioperative patients is really important question for the future. Currently, an informal consultation across multiple perioperative clinical pharmacist service lines occurs where providers fully endorse pharmacy recommendations and defer for preoperative antibiotic and perioperative opioid optimization.

It's always been very challenging to collect data to justify additional resources. It wasn't easy, but if can be done at a community hospital, others can do it, too. The perioperative positions are an integration of clinical and operational activities with clinical predominating. Because of the integration, instead of an additional pharmacist to manage the satellite, the integrated pharmacist can perform a dual role responsibility to generate leverage for more clinical resources, have a wider reach, and assign technical support to collect clinical data while pharmacists clinically support nurses and prescribers. Data-driven outcomes and strong interprofessional collaboration are what strengthened and made it sustainable. This approach, combined with a scholarly approach for contributing to the scientific literature, secures and justifies service expansion by continuously improving processes and assuring better data quality.

Bariatrics - Alyssa Pollock, PharmD, BCPS, Creighton University School of Pharmacy and Health Professions; CHI Health Immanuel, Omaha, NE

Clinical pharmacy has been involved in bariatric surgery for about 10 years, and pharmacy has had a presence in the perioperative process for over 20 years, providing a foundation to establish relationships with bariatric surgeons. One of the clinical pharmacists on service in acute care, who is also a university professor with 50% of time spent at the hospital, performs consults for bariatric surgery patients. A surgical pharmacy is staffed five days per week, and their function is to compound and provide medications for the operative areas. There is no collaborative care process in the surgery or recovery services. An antimicrobial stewardship program exists, but there are no other formal stewardship programs or committees.

Clinical pharmacy has a bariatric consult service through the EHR, and there's no formal CPA. Preoperative consultation consists of meeting with the patient about 2-3 weeks prior to surgery and before starting any type of liquid, low-sugar diet. Once the patient’s medication list is verified as current, the pharmacist reviews to assess the sizes and formulations of solid oral dosage forms. Medications should be smaller than the end of a pencil eraser for 4-6 weeks after surgery. The pharmacist determines whether oral medications can be swallowed whole or if these need to be opened, crushed, or split in half to pass tight surgical sites (sleeves, bands, or GJ anastomosis) [165]. If formulations need to be changed, the original prescriber is contacted for new prescriptions to be dispensed prior to surgery. Patients with diabetes are informed how to adjust diabetes medications when starting the low-cal/low-sugar liquid diet. A plan is devised for when to withhold anticoagulants and immunomodulatory drugs prior to surgery and includes any medications

that will be held or discontinued after postop as a result of surgery (i.e. diuretics, NSAIDs) to avoid unnecessary refills and postoperative complications. Clinical pharmacy also addresses post-discharge VTE prophylaxis prevention is also addressed as needed with pharmacy assistance to assure the patient can has obtained the drug of choice considering their insurance, community pharmacy supply issues, among others.

On the day of surgery, once the patient is admitted, medication reconciliation is performed to minimize postoperative home medications use while inpatient and to ensure administration start time coincides with oral intake. While in the hospital, clinical pharmacy provides daily monitoring to optimizes medications for pain, PONV, and fluid and electrolyte management. While celecoxib is used as part of the postoperative multimodal analgesic regimen, other NSAIDs are avoided due to concerns related to anastomotic leakage. At discharge, medication reconciliation is performed again to address which home medications are to be stopped, continued, and those that have been replaced with a different formulation. Opioid discharge prescribing is limited [166,167]. Discharge orders are reviewed for proton pump inhibitors (PPIs), VTE prophylaxis, and anti-emetics to verify that these medications appear on discharge summary prescribed at the correct dose, for the correct duration, with prescriptions sent to the correct pharmacy. Discharge medication counseling is also conducted to educate the patient on why changes have been made or that may be anticipated due to metabolic changes (i.e. modifications to antidiabetics, antihypertensive on agents, anticoagulants). The pharmacist also ensures the patient knows any pertinent monitoring parameters related to medications changes, and to contact their physician if when problems arise. The goal is to create a safe medication plan for home use.

Post-discharge follow-up includes reminding the patient to schedule appointments with other providers to address medication adjustments. This may include drug levels for psychotropic or epileptic medications, INR monitoring for warfarin, and adjustments to diabetes, hypertension and heart failure regimens. Anti-hypertensive and hypoglycemic medications tend to be the most difficult portion of managing home medications for these patients, mostly because of metabolic changes that begin after surgery [168]. For example, metformin is a large tablet that may need cutting or crushing. If the patient is on an extended-release formulation, there is literature supporting different bioavailability for gastric sleeve versus Roux-en-Y, and problems can be avoided by changing to an immediate release regimen and splitting the tablet. Typically, multivitamins are replaced with a chewable bariatric multivitamin.

In addition to consult activities, the bariatric surgery pharmacist is intricately involved in the protocol design of bariatric surgery order sets in conjunction with surgeons with specific focus on fluid and electrolyte management, pain, PONV and, VTE prophylaxis. The pharmacist also addresses appropriate dosing, duration, and timing of medication administration. The bariatric order sets include an automatic consult to the bariatric surgery pharmacist to perform admission and discharge medication reconciliation to help promote continuity of care and safe mediation use.

Cardiothoracic – William E. Dager, PharmD, BCPS – University of California Davis, Sacramento, CA

Perioperative collaboration with operating room activities grew initially out of my regular activities as a satellite pharmacist in the 1980s with responsibilities to the intensive care units. The OR staff had full control of the drug supply and could pull whatever they wanted from storage bins. There were no restrictions, and the medical record was on paper with no automation or EHR. At that time, I watched some patient cases and learned how much we didn't know about a patient's overall care experience unless we are actually aware of the entire course. Seeing what happens with the patient in the OR provided incredible insights on my ability to practice patient centric precision medicine or individualized care by having a grasp of the big picture and awareness of all potential drivers with their situation. Pharmacists never crossed the OR threshold and what happens there was never taught. It was this black hole where no one

knew what happened and was innovative for a pharmacist to be in the OR and involved with management decisions during a case.

In those early years, I started with letting physicians show and teach me what happens during a case, what to observe, the unexpected challenges faced and the goals for successful procedure outcome. This led to understanding the unique challenges tied to the OR period with patient care and how decisions preoperative and postoperative can impact overall care. These observations and understanding were huge factors allowing me to appreciate at a higher level how to manage patients' medications either in preventing the problem pre-operatively, how we used agents postoperatively or in emergency situations [169,170,171,172,173,174]. We did not have a collaborative practice format when we started clinical bedside management at that time. Peri-operative care evolved as we increased our activities from the satellite operational area to the patient care bedside, then with increased involvement with the services, to peri-operative management primarily focused on anti-microbial selection, selected non-formulary requests for agents to use in the OR and then expanded to intra-op anticoagulation, use of concentrated hemostatic agents or other restricted agents.

With increased pharmacy engagement with the surgical services directly, our practices evolved for services such as trauma, transplant, vascular or cardiothoracic surgery (and occasionally other surgical services in complex situations) to have our bedside presence extend into the OR, huddle or pre-procedural plan for complex cases, or called in to the OR when a crisis situation unfolded to guide or expedite management. For us, it was mostly the cardiac surgery pharmacist or anticoagulation-related specialist who actually entered in the OR, which now has been the case for over 25 years.

What medications they could use in the OR in many cases were formulary-related decisions. One of our first interventions involved the anesthesiologists making infusion drips. Patients would leave the OR and arrive into the intensive care unit with infusion running and a piece of tape with scribble stating "EPI" on the bag and we had to figure out what to admix and hang for the new one. We had no idea what was the concentration. One of the first things we started to change was the mixing method of the anesthesiologists, how infusions were labeled, and what needed to be continued was done correctly. Everything was charted on paper, and the anesthesiology record had to be located to find out when to time antibiotics (while re-assessing primarily postoperative renal function) for a clear transition to the postoperative medication plan. Initially, antibiotic use was one of the first areas of collaborative care [175,176]. We restricted OR antibiotics and that was probably my department's first area of more restrictive practice specific to the type of surgery. Sometimes, only a gram of cefazolin was given to a super obese patient or one that had renal issues. The need was to address how perioperative antibiotic therapy was approached.

The implementation of automated drug dispensing cabinets in the OR allowed pharmacy to track what and where drugs were being used. The integration of the EHR system, EPIC, into the OR including real time anesthesia medication charting and gave us tools to prepare or adjust postoperative medications as well as track how medications were used. The pharmacist could now verify perioperative and selected intraoperative orders (e.g. concentrated clotting factors) during all phases of surgery. Order sets developed between pharmacy, nursing, and physicians facilitated a collaboration on how to organize and optimize selected surgical pathways. Pharmacist verification and ability to schedule created a safety net.

As practice evolved into allowing more bedside clinical activities and the training of student and residents, I began passing on experiences regarding the OR to students and residents. With our visual presence in the OR, even if it just exposed our learners, the surgeons and staff become more familiar with us, and we began exchanging thoughts about the case, either what to do now, what we should have considered perioperatively, postoperatively, and how to improve practice. As the surgeons and anesthesiologists became more familiar with us and how we could successfully facilitate their practice, the word spread via

case reviews, Morbidity and Mortality rounds, and other means, leading them to ask for our assistance, when necessary, in advance or during complex cases. Lately, it comes to us in the format of a page or Vocera message to get a pharmacist into the OR right away, or the attending stating that we will do this case with certain trained individuals including a specific pharmacist in the OR, to weigh in on what we should consider doing if a certain situation developed during surgery.

In the early 90s, I was also tasked with developing an anticoagulation stewardship program [177,178] to deal with challenges and gaps that had been identified. We didn't call it stewardship then but to solve our anticoagulation issues, including bleeding complications in the OR and post operatively, my role began to evolve around how anticoagulation agents were used, adequate reversal, laboratory assessments that evolved into a more precision-based patient management approach. Experience taught me to look at the big picture and all possible variables including what could or did happen in the OR. As my expertise grew with knowledge, we had a problem with cardiac tamponade post-cardiac surgery [179]. I identified the driving issues and lack of the surgeons' understanding of certain labs or drug characteristics (e.g. rebound heparin effect, insufficient protamine reversal and ACT limitations). Theses gaps brought me into the OR to start preventing problems while they were operating. Other examples include keeping on top of the antibiotics to sustain effects in long cases (or promptly starting them if the patient's chest was opened up at the bedside), or adequate options and dosing if resuscitation occurred, or preventing reloading on top of already established antimicrobials (e.g. dose stacking).

As we started getting familiar with other things like heparin-induced thrombocytopenia (HIT), I began to manage anticoagulation and alternative anticoagulation for patients with HIT [180,181]. This required understanding what options we had, if bivalirudin was utilized, the impact of hypothermia periods that can shut down its elimination or impact of ultrafiltration and the change in blood pooling in surgical field pockets that is different. In some cases, HIT was identified during the surgery requiring us to assist in transitioning from heparin to a bivalirudin infusion while on bypass. That evolved into bleeding and thrombosis management, spending time in the OR with complex bleeding and massive trauma cases (and the blood bank running dry) and planning, and implementing, next steps. How do we target hemostatic management in hemophiliacs (including acquired), Jehovah's witness or other unique patients who needed emergency surgeries? No one knew exactly how to do the anticoagulation therapy, so we collaborated to invent strategies right there at the bedside with constant surveillance and minute to minute adjustments. Another is sustaining anticoagulation in patients undergoing abdominal aneurysm repair and insertion of the spinal catheter during surgery.

Interestingly, I published only a few of the many experiences and successes primarily due to lack of time and the demands of patient care. Some are case reports and others case series [182,183,184,185,186,187,188,189]. Some were the first ever experiences reported in managing major bleeding in patients receiving a direct oral anticoagulant (DOAC), limitations in overdoses and use of antidotes, or how to use the DOAC specific assays we had developed to identify levels of anticoagulation present. We also published case reports or concepts for hemodialysis [190,191,192,193,194] using direct thrombin inhibitors in the OR, including how should a direct thrombin inhibitor be reversed when the patient starts bleeding in the OR [195,196,197,198]? As the only pharmacist panel member on the 10th edition for the Chest guidelines on perioperative anticoagulation or thrombosis management for elective procedures, I was able to apply many insights during the process based on experiences [199].

As we developed more clinical services with specific service lines, the pharmacist rounding with the team was able to be more proactive for making specific drug therapy modifications [200,201]. The evolution of implementing CPOE in our hospital gave us the means to order verification to have even a closer presence to what was going on with all perioperative orders. We became more engaged in the decision process, modifying how a specific selected drug was used and eventually we evolved into a practice in which we

were more prospective with how to manage a situation, for example, a Jehovah's witness patient who's not to have blood products, how do we manage that specific situation with its unique aspects?

Having the service-based pharmacist assignment responsibility for all cardiology-related patients in the hospital, which included the preoperative component, in the ICU, in the OR, in the PACU as necessary or transition from the ICU to the floor until discharged along with the thrombosis and hemostasis specialty area provided numerous opportunities to coordinate acer for a plethora of surgical settings and situations. Sometimes things you do have implications for out beyond their ICU stay. In my service line as a service-based pharmacist, I am responsible from the time the patient walked in the hospital to the time they were discharged. More recently, this includes opiate stewardship, which is best initiated in the OR or immediately post procedure. In the anti-thrombosis and hemostasis stewardship role, I was responsible for developing the guidelines for how we managed the peri-operative care for patients on anticoagulation or antiplatelet therapy for either elective procedures, or urgent surgical needs such as massive trauma, or delicate cases involving high risk for thrombosis or bleeding [182,183].

Regarding clinical pharmacist practice in a service-based role for a surgical service line, you have to be ready to engage with care once the patient comes out of the OR. You engage in both short-term, mid-term and long-term management decisions. The short-term for cardiac surgery is looking at the hemodynamics since most of my patients had surgery-induced heart failure and unstable hemodynamics. I’m adjusting medications based on what has just happened hemodynamically and watching selected monitoring parameters, knowing that the cardiovascular system is compromised. I'm assessing their physiology and participating in short term decisions. They may come out on an Impella, ECLS, they're bleeding with high chest tube output or have signs of a potential infection. These are acute issues to which you must pay attention [186,187,188]. In the intermediate stage are more questions on pain or anxiety management, hemodynamic support and tapering off vasoactive agents, extubating and diuresis along with nutrition. For example, when they come out of the OR, I’ll address the entire medication profile, assay results and bedside presentation along with antibiotic and opioid stewardship. How do we allow this patient to get sleep, how do we avoid delirium, are their issues that I need to resolve proactively before they become larger problems. For opiate stewardship, implementing the best non-opioid related pain relief regimen appropriate for the patient situation and using less and less by training the nurses that a little bit of pain is not a bad thing versus having people who are dependent upon these medications. Intravenous acetaminophen orders were stopped after one dose and more cost effective and equivalent options initiated unless very selective situations existed, which I could be part of resolving.

You find the right balance for everything you look at during postoperative recovery. For example, what's going on with the ventilator and other mechanical devices being used. You adapt the pharmacotherapy to those as they wax and wane, or if they go into acute organ failure, you're catching how to reduce the medications. For example, in acute kidney injury (AKI), we adjust the medications bearing in mind that when the patient comes out of the OR, they are often full of fluid and may not be on dialysis. You have to load them up and be effective. Drug elimination in AKI is more rapid than the data generated in CKD, so be cautious not to underdose medications in renal failure and pay attention to expanded volumes of distribution and the need to provide a loading dose if necessary [193]. When AKI is resolving (by seeing increased urine output), doses need to be adjusted upward. Otherwise, you’re playing catchup all the time. The physiology drives your decision-making looking forward to avoid any discharge delays for secondary to unresolved pharmacologic related issues. For example, sometimes the patient is on warfarin. Do we need to have the INR at a certain place at a certain number to discharge for a mechanical mitral valve or left ventricular assist device? I'm advancing the anticoagulation therapy earlier and more aggressively if safe so they can be discharged. You stay on top of the therapy because of your role in transiting to the long-term plan.

Being at the bedside and face to face in discussion with the physicians is vitally important. When you just get on the phone and say well do this and that without really knowing what's going on, you may be approaching care in a limiting silo and focus on particular issues and miss key issues. This can be dangerous because there can be small details that are really important missed. The physicians dealing with things at the bedside doing post-operative care may not the surgeons who had done the procedures. The perioperative staff may not have communicating effectively some of the pharmacologic issues and some therapy issues can be overlooked. To have a really effective patient centric practice, you need to be present when possible, where the action is face to face and develop those relationship and gain the appreciation of the care teams. For example, to understand their dilemma or situation, instead of saying no to a non-formulary drug request and disengage; communicate that we don't have this available under the circumstances, but how can I help you with this situation? Then, you're engaging and being part of the process. To optimize care, you have to have an approachable presence. As you develop that presence and trust, you're going to develop a reputation or a process in which they're going to seek you out and engage you. Then pharmacy is sought out prospectively instead of pharmacy establishing a reactionary perception that it is perceived as a roadblock. For the newer generation practitioners, this seems to be something some struggle with as they increasingly utilize text messaging information or recommendations instead of face to face discussion with the patient, family or bedside care providers. They're reviewing and managing from a room virtually at some other place in the hospital or at an outside site. Key care issues can then be missed that I have appreciated learning only when I see or become informed at the bedside. I chuckle at times when the pharmacist calls to stop antibiotics on the patient whose chest is still open or change IV to oral when the patient has significant residuals and the nurse indicated that everything is coming back up. Remote practice can result in not getting the big picture, assumptions are considered that may not be correct, or even issues involving the pharmacotherapy that should, but not addressed. Good practice and customer service in most patient situations mean being where the action is unfolding and driving prospectively instead of being reactionary. My job in the end is to make the physicians succeed in achieving their goals and the patients to receive optimal care. Years of practice has shown the advantages of looking at the big picture, engaging, and be willing to adapt and advocate for optimal care.

Colorectal – Jenna Lovely, PharmD, BCPS, Mayo Clinic – Rochester, MN

Collaborative care started around 2010 when I piloted collaborative practice agreements (CPAs), first with the colorectal practice and then expanded to other surgical services. Prior to that time, Mayo instituted rounding practices called "fire rounds." The surgeons had a complicated schedule, working in clinic, the OR, and then ward rounding, and so there weren't set times that they would be rounding on their inpatients. A group page to pharmacists would go out when the surgeons arrived on the ward so that pharmacists could join multi-disciplinary rounds, starting around 2006 [17]. To develop the actual collaborative practice, we spent time listening and learning on rounds what their preferences were, what things to look for. We were able to incorporate that learning in the language of the inpatient CPAs [160]. We had CPAs in the outpatient environment for cardiovascular, anticoagulation, and asthma clinics, but this was the first time it had ever been done on the inpatient side. In collaboration with the surgeon-in-chief and three of our pharmacists, we developed and instituted the "contract," which is what Minnesota State law requires. We went through the legal department and since it was the first one on the inpatient side, we spent a lot more time through going through committees and organizational structures to get everything approved. From there, subsequent CPAs were much faster to implement. We started with one colorectal surgeon, three pharmacists and me, then expanded to two surgeons and 16 pharmacists, then to the entire division of nine surgeons. Our hepatobiliary team observed all that was happening, successes spread by word of mouth, and soon our liver specialist was asking, "why can't we have pharmacists on our team?" By natural diffusion, we expanded to the liver group, and then on the entire hepatobiliary, gynecology, and plastics. Then to trauma team and ortho and basically all the other divisions came in subsequent six to nine months to a year-long increments. Mayo now has over 100 different practice specialties using CPAs

with the pharmacists, working at the very top of the license. We use evidence-based practice clinical guidelines that are available nationally and any internally consensus guidelines not covered in national guidelines specific to any particular practice. We identified some very specific things, such as the initiation of full anticoagulation. The surgeons give us the green light for initiation, and from there, we handle all the details. We right fluid boluses for most services other than a carve out for knee replacement as an example. The contract fits on a half a page to a page with major topics covered generically and then, if there's any carve out specific for that particular specialty, we include that in the contract which used to be signed individually. When we first started, the goal was to get people comfortable the idea, so individual surgeons and individual pharmacists signed, because it was a contract between a particular service and pharmacist group. Since then, within probably two years, the chief pharmacy officer signs on behalf of all pharmacists that meet the criteria and the division chief signs on behalf of all physicians in that group. That was a relationship and trust building exercise that we went through. Now it's obviously much more adopted and understood as part of the practice now so nobody questions it. The reason I call that out is because, if a new site is doing this, there is value in building that trust and relationship, and the individual signatures mattered for us to get it started.

In pre-admission clinic, we do not have pharmacists embedded in our surgical clinics. We have multiple clinics in the outpatient practice, and we are not actively involved there at the individual patient level or individual visit, but we do spend a lot of time with the updates for pre-surgical guidelines medication management, what to hold what not to hold. More at the guideline and or order set and patient education level so it's much more global and then individually. We contact patients some services we do phone calls pre-surgical and other. We have a pharmacist taking the medication history prior to surgery so that nearly 100% of our patients have a pharmacist reviewed updated medication list on file prior to them going to the OR [39]. The timing of that is a little different depending on the service.

What has sustained collaborative practice has been the overall partnership where pharmacy took ownership of medication management and the medication list. Every order set and guideline is actively reviewed by a pharmacist. Part of the development and implementation of those standards that have a medication management component relates to research and quality improvement [3,38,58,73,87,146]. Utilizing that data in the next version of whether we need to make a change or not. Pharmacy is readily available to handle logistics including institutional costs to use similarly effective but less costly agents and make that the standard. New medications coming to market are reviewed prior to becoming formulary or restricted or non-formulary. We proactively forecast and plan for all of that, and in real-time shortage situations, we're very systematically making the updates, so that the individual surgeon doesn't have to or team doesn't have to spend time on that. We're doing it as a collective collaborative.

We have a dedicated OR pharmacist team that works in the each of the satellites and most pharmacists are operationally based with a clinical mindset, so at every point in care, they're not only dispensing, they're also understanding all of the clinical aspects as well. All questions and issues are handled by that dedicated OR pharmacist team that covers the OR and satellites in real time by calling into the OR. If surgical team needed to consult the pharmacist, paging and talking with them through the designated phone system facilitates a real time conversation with the anesthesiologist and or the surgical circulator or the surgeon or the CRNA or whoever in the team needs a second set of eyes or advice on something particular. For high risk meds, we have special procedures for example methadone, and then in the PACU the same or pharmacist team would cover. When the patient arrived to the either the ICU, PACU, or a postop unit, an inpatient pharmacist assumes responsibility for care. An immediate medication reconciliation is performed because there's been a care transition. Every medication is reviewed and updated, clinical questions are handled, and 99% of this activity is covered under CPA. If the service needs to be contacted, however, we have the conversation and follow the same procedure. Different units have different rounding patterns, but most of the surgical units have "fire rounds" and in the ICU, it's much more structured at a specific

time. Some of our units have virtual rounding which is a review of the patient profile. They're looking for whether our goals for pain management, VTE prophylaxis, and antibiotic standards are being met [161, 144]. Medication management is handled for the patients, not only in the surgical context but also any of the home medications.

Mayo has antimicrobial stewardship enterprise wide with a committee structure and work groups that's been in place at least over 15 years. Opioid stewardship has evolved after the opioid crisis, and Mayo was doing MME calculations prior to that, but it wasn't a formal program until after the crisis [109]. We were able to drop MMEs by approximately 40 to 50% in every practice area that we've been able to fully implement. We proved that we can actually use multimodal pain control and minimize patients' opioid exposure, not to prevent them from having opioids at all, but to minimize their use, so adverse effects can be minimized. Like opioids, anticoagulation became a formal stewardship program within the last three to five years. From a medication management standpoint, we've been doing the activities of stewardship since I started in 2000. Every year, we've added more rigor into these programs, good pharmaceutical care where the best medication is used at the lowest cost with the best outcome for the patient.

Specific metrics related to pharmacotherapy or medication management across the board for all specialty practices is the overall LOS, surgical LOS which is used as a surrogate. If they're well enough to be out of the hospital, we've met our goals that the patient is well. Complication rates are tracked using the Clavien-Dindo system which tracks with what we were already collecting in our NSQIP program. We measure readmission rates because we want to have a good counterbalance between LOS, readmission LOS, and combined LOS. Pharmaceutical therapy-specific measures have been related antimicrobial prophylaxis for SSI and VTE prophylaxis and usage, both pre and post up and post discharge where applicable. NSAID use is criteria-based to have those scheduled within the first two days after surgery at a minimum. The same with acetaminophen. We tracked that initially, but it's been at 100% so long that it's no longer reported out. Both pre- and postop, we use oral route, not IV, and have a carve-out for a one-time IV dose if the case lasts longer than six hours in the OR.

We also track any particular compliance for the pathway that was particular to that practice area. For example, for bariatrics, we would be tracking that they switched from extended release to immediate release medications and that the vitamins were ordered correctly and that the patient had the very accurate diet pathway. Whereas in colorectal, we would look for diet as tolerated immediately after surgery, for example. If neurosurgery was trying to do something particular with dexamethasone, we would track that particular medication for that practice area. For Whipple procedures, we have a specific assessment for low risk, moderate risk, and high risk for leaks and plan what to look for based on practice guidelines specific to that particular procedure with one or two extra specific measures metrics.

What I would like to share about my collaborative care story at Mayo, the journey that I've been on over these last 20 years, is the acknowledgement that it's been my absolute privilege and responsibility to work with some of the world's best surgeons, to appreciate and honor that I've been able to work with them to create these programs and this partnership that helps us do our work better and has definitely been beneficial from their perspective in a variety of ways, freeing the surgeon from details of pharmacotherapy on a daily basis. They know we've got this covered, and we've also been able to demonstrate, time and time again, that we get great outcomes doing this type of work together that we weren't able to do alone.

Gynecological oncology – Siu-Fun Wong, PharmD, University of California Irvine, Orange, CA

Collaborative work in perioperative care primarily occurred at University of California Irvine (UCI) Medical Center, which began during my UCSF School of Pharmacy senior year clinical clerkship rotations at UCI with both the Gynecology-Oncology and Hematology-Oncology teams. I was fortunate that my

preceptor already had an interprofessional practice and was an excellent role model for collaborative practice. A longitudinal patient care experience in the specialty was subsequently integrated into my UCI residency experience. Upon the completion of my residency, I became the founding Gynecology-Oncology pharmacist of UCI at the request of the gynecological surgical oncologist. At UCI, the gynecology surgeons managed their patients' chemotherapy treatments, which created drug therapy opportunities for a pharmacist to be involved. Rounding with the surgical team daily, I learned the need to adjust my schedule to at-tend early rounds, and the value of understanding the thought process of other healthcare providers to provide relevant concise feedback. This helps to develop relationships, trusts, and teamwork. For example, if the question regarded dosing, a concise answer based on the patient's end organ functions and peri-operative related elements would suffice. However, when at care conference where students and residents were present, attendings relished additional background teaching from the advanced practice pharmacist. Oncology pharmacists also supported the UCI medical oncology team in both inpatient and ambulatory care areas, including developing an infusion center pharmacy at the brand-new UCI Chao Comprehensive Cancer Center from "blueprint to reality" in the late 1980s.

Subsequently, I became in charge of the entire oncology pharmacy program, and UCI was granted National Cancer Institute comprehensive cancer center status. An infusion center pharmacy with two and a half FTE pharmacists and two FTE technicians was created to centralize chemotherapy administration and deliver patient care. The mission was always, no matter which team, send a pharmacist to round with the teams as an integrated inpatient/outpatient model with the infusion center pharmacy as the home base. In those days, chemotherapy orders were handwritten, so order templates and roadmaps were created to minimize error. A patient profile was designed to track relevant information of the patients' chemotherapy because retrieving information from charts was often challenging and time consuming for all health care providers, especially surgeons and oncologists. Attendings and fellows began to rely on these patient profiles, which provided pharmacy the opportunity to further interact with the team and monitor patients' laboratory work and tolerance of their regimens. Pharmacy service using the infusion center pharmacy as a base also included working with nurses for patient education when beginning chemotherapy and reviewing concurrent medication information to assure no new drug-drug interactions occurred [202,203]. Determining risk factors for various adverse events was the first level of focus for supportive care. Often, pharmacy would write the verbal order as a means to actually co-manage the patient. There was no written collaborative practice agreement due to institutional employment arrangements. Each pharmacist earned the trust from the rest of the medical team based on knowing their materials but also recognizing their limits. Both specialists and senior pharmacists earned the right to document these assessments as bona fide patient care activities in the patient's record. This method is how collaborative practice was built without a written agreement. Collaborative practice is a good vehicle to allow some level of independence. However, at the same token, oncology patient care is so complex that it may not be the best approach, because team care for supportive care depends mostly on the relationship with the physician where more independence is permitted in which the advanced practice pharmacist makes a pharmacotherapy decision, writes a verbal order, and documents in the chart note that is sent to the physicians, who reviews and cosigns the order.

In addition to delivering comprehensive cancer chemotherapy care to both gynecologic and medical oncology departments, the pharmacy services provided facilitated connections to the surgical oncology colleagues through pain management and regional chemotherapy administration, and the comprehensive investigational drug service. The latter also brought in the ophthalmology investigators.

Clinical pharmacy also evolved to clinical research, including protocol review and conduction of clinical studies. The cancer center medical director supported me to submit a proposal to SWOG Research Network (formerly known as Southwest Oncology Group), and a pharmacy committee was subsequently created at SWOG in 1994 that exists to this day to support the entire protocol development process.

On becoming a full-time academic for more protected research time, a clinical practice site was needed while at Western University. I teamed with a surgical oncologist in the melanoma clinic at UCI to manage patients enrolled in a melanoma vaccine trial and those receiving high dose interferon as an adjuvant therapy [204]. My role progressed to assess the patient's pain management and make pharmacotherapy recommendations for various medical conditions. I was the first faculty to develop a service at the cancer center, and a written collaborative practice agreement was signed between the School and the Center. Two more practice models with written collaborative practice agreements followed that included clinical research program development at a private medical oncology office and a standalone referral clinic where a pharmacist manages oral chemotherapy for medical and surgical oncologists at Loma Linda University Medical Center [205,206].

As far as other pharmacy perioperative relationships were concerned, at one point, there was an operating room pharmacy and specialty pharmacists were stationed in the recovery room at UCI. Many specialty practice pharmacists have been developed for example, the Intensive Care Unit and Emergency Department pharmacists. All internal medicine teams have a pharmacist assigned. Nurses and the medical staff have learned to appreciate how the pharmacist can complement their work.

This narrative describes an interprofessional pharmacy practice in gynecological and surgical oncology that developed over the last 40 years, culminating in advisement on specialty task forces and tumor board. For an advanced practice pharmacist to be involved in interprofessional care requires being present and ready to engage in promoting the patient's overall well-being. Lean in and be proactive. Be part of the team and document care activities in the patient's medical record. This shows the rest of the team that you are responsible, accountable, and here to take care of the patient. Often, pharmacists give recommendations and play a secondary role in patient care. To me, when providing patient care, one must have face-to-face contact with the patients, not through medical record. This is direct patient care which shows commitment to the patient and the team.

Orthopedics - Dustin Carneal, PharmD, Crystal Clinic Orthopedic Hospital, Akron, OH

The orthopedic service lines at Crystal Clinic include total joint, spine, and plastics. In addition, collaborative care extends to outpatient areas such as sports medicine and hand therapy. About 17,000 surgeries a year are performed. What really propelled the collaborative care movement at Crystal Clinic was its participation in the Medicare shoulder joint replacement program. The goal was to identify and minimize costs through collaboration and teamwork. One gap identified early in the process is now called "Crystal Clear" pathways where a care coordinator is assigned to help a patient navigate perioperative care. Another gap was physical therapy, which led to creation of a total joint class where providers gave specific education points. Clinical pharmacy was invited to provide medication education for rehabilitation in a collaborative model. Discharge counseling became a focus because inappropriate home medications were identified. Then, admission medication reconciliation began as the next process. After this, clinical pharmacy began inpatient rounding daily with internal medicine.

An optimization committee began estimating additional cost savings in the Medicare reimbursement program for readmissions and care after surgery, and identified patients with infections. Pharmacy was key in reviewing patients' medications over the phone preoperatively, ensuring patients withheld the proper medications prior to surgery and adjusting them preoperatively. All patients who had postoperative infections over a three-year period were either under-dosed on antibiotics, had high blood glucose postoperatively, undiagnosed diabetics, or diabetes with suboptimal glycemic control. Pharmacy helped identify those patients and intervened, which led to a pharmacist calling patients preoperatively, staffing the preop and PACU area, and rounding on the floor to assist in hospital and after discharge care.

Pharmacy now educates patients and the team on perioperative glycemic control to optimize insulin dosing towards a targeted blood level [158].

Pharmacy is a steward of all classes of medication. The major drug classes are opioids and antibiotics. For stewardship in the inpatient area, pain management is a major target, and all patients are screened to determine their oral morphine equivalents prior to surgery and ensure they're taking the proper postoperative pain management regimen. Pharmacy ensures that all dosing is appropriate with policies and protocols for NSAIDs and non-opioid analgesics as well as proper medications withheld at admission medication reconciliation. The review is conducted with the patient and with internal medicine.

Pharmacy has two types of collaborative care relationships: (1) institution-based protocols; and (2) individual collaborative practice agreements (CPAs). In the hospital, protocols under the scope of hospital cover all patients, all providers, and all pharmacists. For CPAs, individual pharmacists are credentialled based on additional education and training, years of experience, pharmacy residency, and targeted continuing education and after which are then deemed have the requisite clinical skills. The CPA allows a clinical pharmacist to manage and adjust all drug classes within the scope of the surgery, and write a plan of action. Those pharmacists are credentialled based upon disease state as required in the state of Ohio. CPAs exist for total joint diagnoses with specific providers, and surgeons have signed off for the outpatient setting. Pharmacy sees patients preoperatively and screens for venous thromboembolism risk factors, and monitors home discharges for total joints for bleeds and clots. The shared goal with orthopedic providers is to prevent clots but also prevent bleeds through use of a site-specific screening tool that was developed with our joint surgeons.

The outcome metrics or measures related to the enhanced recovery after surgery are complications, length of stay, and readmissions as well as drug costs and use patterns, for example, IV acetaminophen and sugammadex for neuromuscular blockage reversal. Evaluating the impact of drug therapy on early ambulation is a current focus of collaboration. Liaising with anesthesiology (for neuraxial anesthesia) and physical therapy to coordinate spinal block times and physical therapy as well as calculating and managing postoperative oral morphine equivalent usage improves outcomes and shortens length of stay. For example, identifying patients who have a shorter acting spinal to ambulate on the day of surgery and reducing opioid use on post-op day one, and data to support these practices are forthcoming. Compliance and optimizing or maximizing the use of cefazolin for surgical site prophylaxis and reviewing penicillin allergies to optimize cefazolin use rather than clindamycin.

When cases arise, pharmacy uses a gradual approach so that the physicians know pharmacy is here to collaborate. The approach has been to expand into new areas as situations arise. One of the best ways to sustain collaborative care is start with one or two activities. Under-commit to over-perform. Most recently, a patient with an additive allergy caused us to evaluate all medications to avoid propylene glycol, an additive in liquid acetaminophen and tramadol. Pharmacy identified and filled gaps in care very effectively, adding more staff. For example, when pharmacists went on PTO, those services were suspended. Physicians were contacted and informed about the suspension of services, and missed them so much that they advocated for pharmacy to provide continuous coverage.

Pediatrics – Heather Monk Bodenstab, PharmD, Children’s Hospital of Philadelphia, Philadelphia, PA

Starting in the NICU many years ago, there were a number of professionals trying to manage these patients, surgeons, surgical nurse practitioners, pain management team, pharmacy, among others, but what was interesting was that there were no clinical pathways for these patients. As I began to work with the teams, I noticed that the different types of procedures really warranted different approaches. From a pain perspective, there wasn't a one size fits all. For example, a patient having an inguinal hernia repair is very

different from an exploratory laparotomy for an intestinal issue, a congenital diaphragmatic hernia repair or a tracheostomy placed. This is especially true for an infant with a much older gestational or postnatal post-menstrual age as well with an opioid and or benzodiazepine requirement prior to surgery. It's not appropriate to start babies of different gestational ages on the same morphine opioid milligram/kg/h infusion rate after the procedure. The other thing that comes into play is opioid-sparing therapies with IV acetaminophen, IV ketorolac, and dexmedetomidine. It really came down to looking at the individual patients and figuring out what worked out best for them. However, with that said, in thinking about developing algorithms or pathways for these patients, the best way to do that is to categorize them. I worked with the neonatologists and the surgical nurse practitioners to stratify these patients as opioid naïve and exposed and the type of procedure that they were having. For some patients in the opioid exposed group, it could have been something as simple as adding on scheduled acetaminophen and ketorolac. Conversely, if you're having a more intricate surgical procedure, a 20% increase in their continuous opioid infusion rate is needed in conjunction with opioid-sparing therapies. The opioid naïve patient will also utilize opioid sparing therapies, but as compared to the opioid naive patient, where a lower starting dose of morphine infusion is adequate.

Clinical pain management pathways started with neonates to improve post-operative pain scores, prevent adverse events and prevent high-alert medication errors. derive a better dosing schedule postoperatively. If opioid exposed patients were started on a dose for a naïve patient, withdraw symptoms and uncontrolled pain would occur that would complicate the postoperative course. Pathways were constructed and clinical pharmacy consults were instituted. If the patient was going to surgery, the team would request a clinical pharmacy consult. I would evaluate the patient, accounting for their gestational and postnatal age, their underlying conditions, respiratory status (intubated vs. extubated), and current opioid/benzodiazepine/dexmedetomidine requirements, previous procedures and what procedure(s) they were having. If it was something that I wasn't familiar with, I would discuss with the surgical team, or with the surgical nurse, the surgeons, the neonatologist, and the nurse practitioners to get a better idea of what to expect. Will they be intubated in the OR or prior to the procedure, and are they coming back intubated? All of this plays a part in pain as well as sedation management, and the level of sedation is also important. We've noticed that pain score goals without evaluating the level of sedation. Pharmacy worked very closely with neonatology as well as nursing and other management within the hospital to create a hospital wide initiative, where we instituted the use of state behavioral scale (SBS) goals for intubated patients. For those patients who were intubated, understanding the goal the level of sedation allowed us to wean medications using both pain and SBS scores and have goals that are discussed daily on rounds to optimize therapy. This initiative was an excellent example of collaboration between medical staff, nursing staff, and pharmacy. In EPIC EHR, a graph of pain and SBS scores gives you the data to suggest that the patient may be ready for a wean. Additionally, having active discussions surrounding the continued need for post-operative IV access (central or peripheral) allowed us to optimize outcomes for patients. or a switch to oral medications. It was a shared multi-disciplinary approach that had significant benefit for our patients. Another massive component of perioperative care for patient safety was doing an oral sedation wean to eliminate a decrease central line associated risks like central line-associated blood stream infection (CLABSI) and clot formation or a PIV infiltrate to decrease the risk for CLABSI infection or a PIV infiltrate.

Converting routes of administration was a crucial function because the team may not do the right conversion leading to a higher risk of error. I worked in conjunction with neonatologists, surgery and surgical nurse practitioners to develop a clinical pathway related to route changes prior to a procedure as well as when to hold feeds. The pathway decreased a lot of confusion and medication errors in the perioperative period [207].

How can you as a pharmacist build a relationship that garners these types of opportunities to improve patient care? Being present is number one, being available for the medical and nursing team, and being

willing to help is what sustains collaborative care. When they would see that you took the time to write a plan, and it wasn't a copy and paste, you develop credibility and respect. You are literally explaining everything so that there's not that panic moment in the middle of the night about what to do (e.g. how to increase, what to add and when, monitoring parameters, IV compatibility, etc.). If they needed additional guidance for a very difficult patient the team knew that they could come to and count on you for a thoughtful recommendation, that was the most important thing for maintaining those collaborative relationships. Being present and listening. Asking questions if you're not familiar with the procedure.

Anecdotally, I can tell you that when we started using scheduled acetaminophen in any post-operative patient and IV ketorolac in patients greater than 44 weeks postmenstrual age, opioid requirements went own drastically.

For drug stewardship, I worked with surgery, neonatology, and infectious disease on the perioperative antibiotic pathway, deciding what medications were needed and for how long, based on the individual procedures and gestational age-appropriate dosing [208,209,210,211]. For example, eliminating ceftriaxone to avoid development of crystal deposits in organs if it's administered within 48 hours of a calcium-containing solution, like TPN and lactated Ringer's solution.

To develop collaborative practice, engage everyone on the team. Start out small and be patient-focused. Whoever is managing those patients on that team because they're the ones that are going to be able to tell you the problems that they're having [212,213,214,215]. If you voice an interest in learning about those problems, they will tell you what are their major concerns. Ask for their input, especially engaging the nursing staff, not just nursing leadership, but bedside nurses, the seasoned ones as well as less senior ones [216]. You'll learn that their practice styles are very different. Sometimes, surgeons can be a little intimidating, but once you're voicing a concern and you can get them to sit down, they'll talk with you.

Transplant – Tracy M. Sparkes, PharmD, BCTXP, University of Maryland Medical Center, Baltimore, MD

Collaborative services in transplant began in the 1990s and, initially, as the transplant program grew, a pharmacist was stationed in the PACU in order to have access to patient's charts and became highly involved. The pharmacist became very instrumental for medication management by following patients, being outcomes focused, and helping to reduce errors. That initial foray grew into collaborative practice somewhere around 1997, initially in abdominal transplant services, specifically kidney and liver transplant. Overall as an institution, there exists an ever-growing collaborative environment, with numerous collaborative practices in place. The pharmacy team manages antibiotic therapies, such as vancomycin and aminoglycosides encompassing the peri-operative period for surgical prophylaxis. Pharmacists also manage the majority of anticoagulation, including warfarin as well as DOAC therapies, identifying withholding and re-initiation times.

After completing my PGY1 and PGY2 pharmacy residencies, the already established collaborative practice really drew me to the position, especially the idea that I could really be deeply involved in the patient's care. When I joined the team, collaborative services with kidney, pancreas, and liver transplant surgeons and medical team providers were ongoing. A collaborative practice agreement (CPA) with the transplant medicine service also was in place for patients who are further out from their transplant, as well as off-service consult patients [162]. Transplant pharmacists manage immunosuppressants and related medications for the duration of hospitalization and also in the kidney transplant clinic [217,218]. Medications managed by transplant pharmacists include but are not limited to induction immunosuppression (methylprednisolone, rabbit anti-thymocyte globulin, alemtuzumab, and basiliximab) maintenance immunosuppression (tacrolimus, mycophenolate, prednisone, etc.), opportunistic infection prophylaxis, and medications for the treatment of rejection [219]. Collaborative protocols or pharmacy to

dose protocols have credentialing processes that involve providing supporting evidence of background training, residency training and other education specific to transplant, and within transplant there is a competency in place called the "CPA exam" to qualify for collaborative practice.

The infectious diseases pharmacy team has had a long-standing practice and involvement in antimicrobial stewardship and all pharmacists are able to adjust IV to PO and duplicate therapies.

Collaboration between physicians on surgical and medical teams with clinical pharmacy specialists and clinical pharmacists occurs throughout the hospital. There is no formalized perioperative or pre-operative clinic for transplant pharmacy, but the different specialty lines may have their own ad hoc involvement. For solid organ transplants from living donors, transplant pharmacy helps prepare the patient for surgery by identifying medication management plans ahead of time.

What has sustained collaborative agreements are the relationships that pharmacy has established with our surgeons and medical teams. These colleagues rely on pharmacy for medication management, and the protocols and practices are reevaluated periodically to continually develop and optimize new areas. This keeps all parties engaged and helps to ensure enough resources are allocated. For example, CPA services have been expanded to heart and lung transplant patients [220]. Sustaining that additional workload when people leave the institution or go on vacation is a consideration when thinking about collaborative practice development.

Enhanced recovery is a growing area for transplant service lines [221]. Several transplant pharmacy colleagues have been very involved in development of these protocols with a focus on the medication aspect for pancreas and kidney transplant patients. The next area for development and implementation will be kidney and liver transplant living donors. Pharmacists monitor opioid use as a metric, as well as length of stay, both in and outside the ICU.

Across the institution, collaborative practices have developed from great working relationship between pharmacists, other disciplines, and various teams. The pharmacy department expanded into heart and lung transplant, as well as coverage of transplant clinics to enhance education and medication management, to set patients up for success with realistic expectations about the transplant process and life afterwards. Transplant pharmacists have a rotational model through many inpatient and outpatient service lines. Finally, the most recent expansion was longitudinal nephrology clinic follow up for kidney transplant patients as it is the largest clinic [222].

Vascular - Ryan M. Cassity, PharmD, Mayo Clinic – Rochester, MN

I came to Mayo after matching to the pharmacy residency program and spent a year in the PGY1 general practice then continued into a PGY2 critical care. After completion, there was an opening in Mayo's thoracic and vascular ICU. I have been assigned here a little over 20 years. Our service is called the critical care service or CCS service, an 18-bed ICU. We've continued to grow the practice which started off with rounding with the service. Since that time, we've essentially trained all but a handful of our consultant staff as they come through as junior residents and then seniors or fellows and eventually consultant staff. In the Mayo model, the consultant staff is the attending physicians. All attending physicians are anesthesia trained, and they are required to rotate through thoracics, so we get to spend quality time with them.

Our collaborative care model has evolved over the years, and we've developed excellent relationships with the providers. I think that trust that we've gained from them has allowed us to expand our model. Prior to CPAs, we entered verbal orders with attending co-signatures or discussed on rounds to help the residents generate orders. Pharmacy is one of the few consistent departmental staffs on the unit for a long period of time. A consultant staff might work the ICU in February, but might not be here again until October, so they

really look to us as a stabilizing influence on the unit to know the changes, whether it's processes, a guideline change, medication changes, surgeon preference changes. We're that stable group that is able to provide feedback, and collaborative practice has flourished. We can change any medication that we deem appropriate from our clinical standpoint. However, if it is something that we know is going to affect patient care, such as restarting home medications, this would be a discussion on morning rounds. We have liberty to adjust medication dosages based on renal or cognitive function, intravenous to oral route as we deem appropriate as long as the gut is functioning, or we are able to resume home medications, such as an SSRI or levothyroxine. We order laboratory for drug monitoring. For total parenteral nutrition patient, we order triglyceride and phosphorus levels or CMP if they're not in the morning labs.

Postoperative order sets have been designed specifically for the vascular practice, though the vascular surgeons have preferences that are different among their colleagues. The CCS team is considered the primary team and only writes orders on patients in the CCS unit. As far as specific medication management for vascular patients, we have an order set that includes prn medications for tight blood pressure control which would include IV labetalol and hydrazine with parameters for heart rate and blood pressure. Perioperative antibiotics are monitored for compliance with guidelines and CMS, making sure that we discontinue 24 hours from the last coverage after the last dose in the OR. A couple of things that pharmacists do that are not on those order sets, such as stress ulcer prophylaxis and DVT prophylaxis. These are addressed on all these patients every day.

A major revascularization surgery patient is at risk for lower extremity or even upper extremity weakness due to the lack of blood flow. In these cases, blood pressure augmentation protocols are activated to keep mean arterial pressure as high as 90 with norepinephrine. It’s pretty amazing what the vascular surgeons can do, where they can custom make an arterial graft with specific holes that allow the hepatic artery or renal arteries to circulate the mesenteric area.

This is a lesson for pharmacy residents when they rotate through that all these patients are at risk for renal injury after the procedure because, in order to fix the aneurysm, often the aorta is clamped above and below the aneurysm. Major organ and lower extremity ischemia can occur, especially when bypass is needed in open procedures for abdominal aneurism repair, where the bulge can be anywhere from the aortic arch to the femoral arteries. Major vascular surgery procedures are abdominal aneurysms and depending on where they're at in the vessel will determine patient outcomes and the side effects of the surgery. When a Gortex graft is placed, it's a foreign material that can get infected like a prosthetic joint or valve, and it’s a huge issue and hard to treat because graft removal is not easy. Usually, the patient is on antibiotics for weeks at a time, sometimes in the ICU for weeks or months at a time. Source control is very tough. At the last resort the infected graft is removed and that leaves scarring

To sustain collaborative practice, the number one thing is to keep the trust of the providers by providing value through medication management. I am a member of the ICU Management Committee, and readmissions, length of intubation, and hospital-acquired infections are tracked. If patients are readmitted to the ICU as a “bounce back” within 24 hours, the committee will figure out why the patient looked good for discharge and why they bounced back. What was missed. Through this committee, pharmacy reviews all the protocols that will be used in the CCS unit and provide feedback before it goes into production.

For anyone who's trying to develop a collaborative practice in a perioperative area, develop and maintain trust and provide value to the service. You have to provide something that other healthcare providers do not contribute and perform at a high level. Do no harm and do your best for the patient. If you're going to practice in an ICU environment, it's very beneficial to get additional training such as a critical care residency. There's a huge gap in knowledge between learning something from a book and then applying it to the real practice. On rounds, everyone has their say, everyone has their input and sometimes the medical

residents, especially in their first year, can almost be too trusting. I use that as a teaching point. Question me, think about it systematically and logically so that you understand why I made a specific recommendation or why we're doing something specifically with this patient's MAP goal of 90 instead of the normal 65 mm Hg. Just because I say something and have 20 plus years of experience doesn't make it right.

References (numbering based on appearance in main text)

3. Lovely, J.K.; Hyland, S.J.; Smith, A.N.; Nelson, G.; Ljungqvist, O.; Parrish, R.H. 2nd. Clinical pharmacist perspectives for optimizing pharmacotherapy within Enhanced Recovery After Surgery (ERAS®) programs. *Int. J. Surg.* **2019**, *63*, 58-62. https://doi.10.1016/j.ijsu.2019.01.006.
17. Wilson, J.W.; Oyen, L.J.; Ou, N.N.; McMahon, M.M.; Thompson, R.L.; Manahan, J.M.; Graner, K.K.; Lovely, J.K.; Estes, L.L. Hospital rules-based system: The next generation of medical informatics for patient safety. *Am. J. Health Syst. Pharm.* **2005**, *62*, 499-505. https://doi.10.1093/ajhp/62.5.499.
38. Grass, F.; Lovely, J.K.; Crippa, J.; Mathis, K.L.; Hübner, M.; Larson, D.W. Early acute kidney injury within an established enhanced recovery pathway: Uncommon and transitory. *World J. Surg.* **2019**, *43*, 1207-1215. https://doi.10.1007/s00268-019-04923-1.
39. Schreier, D.J.; Lovely, J.K. Optimizing clinical monitoring tools to enhance patient review by pharmacists. *Appl. Clin. Inform.* **2021**, *12*, 621-628. https://doi.10.1055/s-0041-1731341.
58. Kelley, S.R.; Wolff, B.G.; Lovely, J.K.; Larson, D.W. Fast-track pathway for minimally invasive colorectal surgery with and without alvimopan (Entereg)™: Which is more cost-effective? *Am. Surg.* **2013**, *79*, 630-633.
73. Kalogera, E.; Bakkum-Gamez, J.N.; Weaver, A.L.; Moriarty, J.P.; Borah, B.J.; Langstraat, C.L.; Jankowski, C.J.; Lovely, J.K.; Cliby, W.A.; Dowdy, S.C. Abdominal incision injection of liposomal bupivacaine and opioid use after laparotomy for gynecologic malignancies. *Obstet. Gynecol.* **2016**, *128*, 1009-1017. https://doi.10.1097/AOG.0000000000001719.
74. Hyland, S.J.; Deliberato, D.G.; Fada, R.A.; Romanelli, M.J.; Collins, C.L.; Wasielewski, R.C. Liposomal Bupivacaine Versus Standard Periarticular Injection in Total Knee Arthroplasty With Regional Anesthesia: A Prospective Randomized Controlled Trial. *J. Arthroplasty.* **2019**, *34*, 488-494. https://doi.10.1016/j.arth.2018.11.026.
87. Merchea, A.; Lovely, J.K.; Jacob, A.K.; Colibaseanu, D.T.; Kelley, S.R.; Mathis, K.L.; Spears, G.M.; Huebner, M.; Larson, D.W. Efficacy and outcomes of intrathecal analgesia as part of an enhanced recovery pathway in colon and rectal surgical patients. *Surg. Res. Pract.* **2018**, *2018*, 8174579. https://doi.10.1155/2018/8174579.111. Hyland, S.J.; Kramer, B.J.; Fada, R.A.; Lucki, M.M. Clinical pharmacist service associated with improved outcomes and cost savings in total joint arthroplasty. *J. Arthroplasty.* **2020**, *35*, 2307-2317.e1. https://doi.10.1016/j.arth.2020.04.022.
109. Gazelka, H.M.; Clements, C.M.; Cunningham, J.L.; Geyer, H.L.; Lovely, J.K.; Olson, C.L.; Philpot, L.M.; Porter, S.B.; Witt, T.J.; Zavaleta, K.W.; Habermann, E.B. An institutional approach to managing the opioid crisis. *Mayo Clin. Proc.* **2020**, *95*, 968-981. https://doi.10.1016/j.mayocp.2019.11.019.
111. Hyland, S.J.; Kramer, B.J.; Fada, R.A.; Lucki, M.M. Clinical pharmacist service associated with improved outcomes and cost savings in total joint arthroplasty. *J. Arthroplasty* **2020**, *35*, 2307–2317.e1. https://doi.org/10.1016/j.arth.2020.04.022.
112. Hyland, S.J.; Brockhaus, K.K.; Vincent, W.R. 3rd; Spence, N.Z.; Lucki, M.M.; Howkins, M.J.; Cleary, R.K. Perioperative pain management and opioid stewardship: A practical guide. *Healthcare (Basel).* **2021**, *9*, 333. https://doi.10.3390/healthcare9030333.
144. Morgenthaler, T.I.; Lovely, J.K.; Cima, R.R.; Berardinelli, C.F.; Fedraw, L.A.; Wallerich, T.J.; Hinrichs, D.J.; Varkey, P. Using a framework for spread of best practices to implement successful venous thromboembolism prophylaxis throughout a large hospital system. *Am. J. Med. Qual.* **2012**, *27*, 30–38. https://doi.10.1177/1062860611411518.
146. Danelich, I.M.; Lose, J.M.; Wright, S.S.; Asirvatham, S.J.; Ballinger, B.A.; Larson, D.W.; Lovely, J.K. Practical management of postoperative atrial fibrillation after noncardiac surgery. *J. Am. Coll. Surg.* **2014**, *219*, 831–841. https://doi.10.1016/j.jamcollsurg.2014.02.038.
157. Hyland, S.J.; Kusumi, R.K.; Lopez, L.F.; Kramer, B.J.; Fada, R.A.; Mohan, VS.; Rodgers, J.K.L.; Lucki, M.M. Antimicrobial Stewardship in Total Joint Arthroplasty: Outcomes of a Collaborative Program Implementation. J. *Amer. Acad. Orthop. Surg*. **2022**, June 30, 10.5435/JAAOS-D-21-00722. https:// doi.10.5435/JAAOS-D-21-00722
158. Donihi, A.C.; Moorman, J.M.; Abla, A.; Hanania, R.; Carneal, D.; MacMaster, H.W. Pharmacists' role in glycemic management in the inpatient setting: An opinion of the endocrine and metabolism practice and research network of the American College of Clinical Pharmacy. *J. Am. Coll. Clin. Pharm.* **2019**, *2*, 167-176. https://doi.10.1002/jac5.1041.

160. Lovely, J.K.; Larson, D.W.; Quast, J.M. A clinical practice agreement between pharmacists and surgeons streamlines medication management. *Jt. Comm. J. Qual. Patient Saf.* **2014**, *40*, 296-302. https://doi.10.1016/s1553-7250(14)40039-4.
161. Larson, D.W.; Lovely, J.K.; Welsh, J.; Annaberdyev, S.; Corning Coffey, C.; Murray, B.; Rose, D.; Prabhakar, L.; Torgenson, M.; Dankbar, E.; Larson, M.V.; Mayo Clinic Care Network Colon Rectal Surgery Enhanced Recovery Collaborative Group. A collaborative for implementation of an evidence-based clinical pathway for enhanced recovery in colon and rectal surgery in an affiliated network of healthcare organizations. Jt. Comm. J. Qual. Patient Saf. 2018, 44, 204–211. https://doi.10.1016/j.jcjq.2017.08.007.
165. Smith, A.N.; Henriksen, B.; Cohen, A. Pharmacokinetic considerations in Roux-en-Y gastric bypass patients. *Am. J. Health Syst. Pharm.* **2011**, *68*, 2241-2247. https://doi.10.2146/ajhp100630.
166. Smith, A.N. Opioid prescribing patterns at discharge for surgical patients. *Int. Anesthesiol. Clin.* **2020**, *58*, 50-56. https://doi.10.1097/AIA.0000000000000274.
167. Cohen, A.R.; Smith, A.N.; Henriksen, B.S. Postoperative opioid requirements following Roux-en-Y gastric bypass in patients receiving continuous bupivacaine through a pump system: A retrospective review. *Hosp. Pharm.* **2013**, *48*, 479-483. https://doi.10.1310/hpj4806-479.
168. Howard, M.L.; Steuber, T.D.; Nisly, S.A. Glycemic Management in the Bariatric Surgery Population: A Review of the Literature. *Pharmacotherapy.* **2018**, *38*, 663-673. https://doi.10.1002/phar.2120.
169. Dager, W.E. Achieving optimal antiarrhythmic therapy in advanced cardiac life support. *Crit. Care Med.* **2006**, *34*, 1825-1826. https://doi.10.1097/01.CCM.0000219375.94748.C4.
170. Dager, W.E. Developing a management plan for oral anticoagulant reversal. *Am. J. Health Syst. Pharm.* **2013**, *70*, S21-31. https://doi.10.2146/ajhp130042.
171. Kulig, C.E.; Roberts, A.J.; Rowe, A.S.; Kim, H.; Dager, W.E. INR Response to Low-Dose Vitamin K in Warfarin Patients. *Ann. Pharmacother.* **2021**, *55*, 1223-1229. https://doi.10.1177/1060028021993239.
172. Dager, W.E. Considerations for drug dosing post coronary artery bypass graft surgery [ed]. *Ann. Pharmacother.* **2008**, *42*, 421-424. https://doi.10.1345/aph.1L009.
173. Dager, W.E. Warfarin for venous thromboembolism prophylaxis after elective hip or knee arthroplasty: exploring the evidence, guidelines, and challenges remaining. *Ann. Pharmacother.* **2012**, *46*, 79-88. https://doi.10.1345/aph.1P626.
174. Nishijima, D.K.; Dager, W.E.; Schrot, R.J.; Holmes, J.F. The Efficacy of Factor VIIa in Emergency Department Patients with Warfarin Use and Traumatic Intracranial Hemorrhage. *Acad. Emerg. Med.* **2010**, *17*, 244-251. https://doi.10.1111/j.1553-2712.2010.00666.x.
175. Heintz, B.H.; Matzke, G.R.; Dager, W.E. Antimicrobial dosing concepts and recommendations for critically ill adult patients receiving continuous renal replacement therapy or intermittent hemodialysis. *Pharmacotherapy.* **2009**, *29*, 562-577. https://doi.10.1592/phco.29.5.562.
176. Hoff, B.M.; Maker, J.H.; Dager, W.E.; Heintz, B.H. Antibiotic Dosing for Critically Ill Adult Patients Receiving Intermittent Hemodialysis, Prolonged Intermittent Renal Replacement Therapy, and Continuous Renal Replacement Therapy: An Update. *Ann. Pharmacother.* **2020**, *54*, 43-55. https://doi.10.1177/1060028019865873.
177. Dager, W.E.; Gulseth, M.P. Implementing anticoagulation management by pharmacists in the inpatient setting. *Am. J. Health Syst. Pharm.* **2007**, *64*, 1071-1079. https://doi.10.2146/ajhp060133.
178. Nutescu, E.A.; Spinler, S.A.; Wittkowsky, A.; Dager, W.E. Low-molecular-weight heparins in renal impairment and obesity: available evidence and clinical practice recommendations across medical and surgical settings. *Ann. Pharmacother.* **2009**, *43*, 1064-1083. https://doi.10.1345/aph.1L194.
179. Dager, W.E.; Sanoski, C.A.; Wiggins, B.S.; Tisdale, J.E. Pharmacotherapy Considerations in Advanced Cardiac Life Support. *Pharmacotherapy.* **2006**, *26*, 1703-1729. https://doi.10.1592/phco.26.12.1703.
180. Dager, W.E.; White, R.H. Treatment of heparin-induced thrombocytopenia. *Ann. Pharmacother.* **2002**, *36*, 489-503. https://doi.10.1345/aph.1A204.
181. Dager, W.E.; White, R.H. Pharmacotherapy of heparin-induced thrombocytopenia. *Expert Opin. Pharmacother.* **2003**, *4*, 919-940. https://doi.10.1517/14656566.4.6.919.
182. Dager, W.E.; Gosselin, R.C.; Owings, J.T: Argatroban therapy for antithrombin deficiency and mesenteric thrombosis: A case report and review of the literature. *Pharmacotherapy.* **2004**, *24*, 659-663. https://doi.10.1592/phco.24.6.659.34745.
183. Dager, W.E.; Regalia, R.; Williamson, D.; Gosselin, R.C.; Tharrett, R.S.; White, R.H.; Albertson, T.E. Reversal of elevated International Normalized Ratios and Bleeding with low-dose Recombinant Activated Factor VIIa in patients receiving warfarin. *Pharmacotherapy.* **2006**, *26*, 1091-1098. https://doi.10.1592/phco.26.8.1091.
184. Dager, W.E. Aminoglycosides in Extended Duration Daily (EDD) Hemodialysis: Pharmacokinetic Observations. *Ann. Pharmacother.* **2006**, *40*, 783-784. https://doi.10.1345/aph.1G420.

185. Steele, A.P.; Lee, J.A.; Dager, W.E. Incomplete dabigatran reversal with idarucizumab. *Clin. Toxicol. (Phila).* **2018**, *56*, 216-218. https://doi.10.1080/15563650.2017.1349911.
186. Nagle, E.L.; Tsu, L.V.; Dager, W.E. Bivalirudin for anticoagulation during hypothermic cardiopulmonary bypass and recombinant factor VIIa for iatrogenic coagulopathy. *Ann. Pharmacother.* **2011**, *45*, e47. https://doi.10.1345/aph.1P785.
187. Nagle, E.L.; Dager, W.E.; Duby, J.J.; Roberts, A.J.; Kenny, L.E.; Murthy, M.S.; Pretzlaff, R.K. Bivalirudin in pediatric patients maintained on extracorporeal life support. *Pediatr. Crit. Care Med.* **2013**, *14*, e182-188. https://doi.10.1097/PCC.0b013e31827200b6.
188. Walker, E.A.; Roberts, A.J.; Louie, E.L.; Dager, W.E. Bivalirudin Dosing Requirements in Adult Patients on Extracorporeal Life Support with or without Continuous Renal Replacement Therapy. *ASAIO J.* **2019**, *65*, 134-138. https://doi.10.1097/MAT.0000000000000780.
189. Kulig, C.E.; Schomer, K.J.; Black, H.B.; Dager, W.E. Activated Partial Thromboplastin Time Versus Anti-Factor Xa Monitoring of Heparin Anticoagulation in Adult Venoarterial Extracorporeal Membrane Oxygenation Patients. *ASAIO J.* **2021**, *67*, 411-415. https://doi.10.1097/MAT.0000000000001246.
190. Pon, T.K.; Dager, W.E.; Roberts, A.J.; White, R.H. Subcutaneous enoxaparin for therapeutic anticoagulation in hemodialysis patients. *Thromb. Res.* **2014**, *133*, 1023-1028. https://doi.10.1016/j.thromres.2014.03.036.
191. Walker, E.A.; Dager, W.E. Bridging with Tirofiban during Oral Antiplatelet Interruption: A Single-Center Case Series Analysis Including Patients on Hemodialysis. *Pharmacotherapy.* **2017**, *37*, 888-892. https://doi.10.1002/phar.1956.
192. Dager, W.E. Filtering out important considerations with developing drug dosing regimens in extended duration dialysis (EDD). *Crit. Care Med.* **2006**, *34*, 240-241. https://doi.10.1097/01.ccm.0000190903.21395.
193. Dager, W.E. What are the important drug use errors in dialysis patients? Pharmacokinetic and pharmacodynamic principles. *Semin. Dial.* **2010**, *23*, 466-469. https://doi.10.1111/j.1525-139x.2010.00773.x.
194. Dager, W.E.; King, J.H. Aminoglycosides in intermittent hemodialysis: pharmacokinetics with individual dosing. *Ann. Pharmacother.* **2006**, *40*, 9-14. https://doi.10.1345/aph.1G064.
195. Dager, W.E.; Roberts, A.J.; Nishijima, D.K. Effect of low and moderate dose FEIBA to reverse major bleeding in patients on direct oral anticoagulants. *Thromb. Res.* **2019**, *173*, 71-76. https://doi.10.1016/j.thromres.2018.11.009.
196. Dager, W.E. Using prothrombin complex concentrates to rapidly reverse oral anticoagulant effects. *Ann. Pharmacother.* **2011**, *45*, 1016-1020. https://doi.10.1345/aph.1Q288.
197. Dager, W.E.; Gosselin, R.C.; Roberts, A.J. Reversing dabigatran in life-threatening bleeding occurring during cardiac ablation with factor eight inhibitor bypassing activity. *Crit. Care Med.* **2013**, *41*, e42-46. https://doi.10.1097/CCM.0b013e31827caaa3.
198. Van Cott, E.M.; Roberts, A.J.; Dager, W.E. Laboratory Monitoring of Parenteral Direct Thrombin Inhibitors. *Semin. Thromb. Hemost.* **2017**, *43*, 270-276. https://doi.10.1055/s-0036-1597297.
199. Douketis, J.D.; Spyropoulos, A.C.; Murad, M.H.; Arcelus, J.I.; Dager, W.E.; Dunn, A.S.; Fargo, R.A.; Levy, J.H.; Samama, C.M.; Shah, S.H.; Sherwood, M.W.; Tafur, A.J.; Tang, L.V.; Moores, L.K. Perioperative Management of Antithrombotic Therapy: An American College of Chest Physicians Clinical Practice Guideline. *Chest.* **2022**, *S0012-3692*, 01359-9. https://doi.10.1016/j.chest.2022.07.025.
200. Dager, W.E.; Inciardi, J.F.; Howe, T.L. Estimating phenytoin concentrations by the Sheiner-Tozer method in adults with pronounced hypoalbuminemia. *Ann. Pharmacother.* **1995**, *29*, 667-670. https://doi.10.1177/106002809502907-802.
201. Dager, W.E. Phenytoin assay errors in uremia. *Ann. Pharmacother.* **2002**, *36*, 939-940. https://doi.10.1177/106002800203600503.
202. Marcath, L.A.; Finley, C.M.; Wong, S.F.; Hertz, D.L. Drug-drug interactions in subjects enrolled in SWOG trials of oral chemotherapy. *BMC Cancer.* **2021**, *21*, 324. https://doi.10.1186/s12885-021-08050-w.
203. Marcath, L.A.; Coe, T.D.; Shakeel, F.; Reynolds, E.; Bayuk, M.; Haas, S.; Redman, B.G.; Wong, S.F.; Hertz, D.L. Improvement Initiative to Develop and Implement a Tool for Detecting Drug-Drug Interactions During Oncology Clinical Trial Enrollment Eligibility Screening. *J. Patient Saf.* **2021**, *17*, e28-e34. https://doi.10.1097/PTS.0000000000000772.
204. Wong, S.F.; Jakowatz, J.G.; Taheri, R. Management of hypertriglyceridemia in patients receiving interferon for malignant melanoma. *Ann. Pharmacother.* **2004**, *38*, 1655-1659. https://doi.10.1345/aph.1D546.
205. Wong, S.F.; Bounthavong, M.; Nguyen, C.P.; Chen, T. Outcome Assessments and Cost Avoidance of an Oral Chemotherapy Management Clinic. *J. Natl. Compr. Canc. Netw.* **2016**, *14*, 279-285. https://doi.10.6004/jnccn.2016.0033.

206. Wong, S.F.; Bounthavong, M.; Nguyen, C.; Bechtoldt, K.; Hernandez, E. Implementation and preliminary outcomes of a comprehensive oral chemotherapy management clinic. *Am. J. Health Syst. Pharm.* **2014**, *71*, 960-965. https://doi.10.2146/ajhp130278.
207. McIntyre, C.M.; Monk, H.M. Medication absorption considerations in patients with postpyloric enteral feeding tubes. *Am. J. Health Syst. Pharm.* **2014**, *71*, 549-556. https://doi.10.2146/ajhp130597.
208. Wade, K.C.; Monk, H.M. New antifungal and antiviral dosing. *Clin. Perinatol.* **2015**, *42*, 177-194, x. https://doi.10.1016/j.clp.2014.10.010.
209. Gannon, A.W.; Monk, H.M.; Levine, M.A. Cinacalcet monotherapy in neonatal severe hyperparathyroidism: a case study and review. *J. Clin. Endocrinol. Metab*. **2014**, *99*, 7-11. https://doi.10.1210/jc.2013-2834.
210. Lawrence, K.M.; Hedrick, H.L.; Monk, H.M.; Herkert, L.; Waqar, L.N.; Hanna, B.D.; Peranteau, W.H.; Rintoul, N.E.; Hopper, R.K. Treprostinil Improves Persistent Pulmonary Hypertension Associated with Congenital Diaphragmatic Hernia. *J. Pediatr*. **2018**, *200*, 44-49. https://doi.10.1016/j.jpeds.2018.04.052.
211. Rustico, S.E.; Kelly, A.; Monk, H.M.; Calabria, A.C. Calcitriol treatment in metabolic bone disease of prematurity with elevated parathyroid hormone: A preliminary study. *J. Clin. Transl. Endocrinol*. **2014**, *2*, 14-20. https://doi.10.1016/j.jcte.2014.12.001.
212. Jensen, E.A.; White, A.M.; Liu, P.; Yee, K.; Waber, B.; Monk, H.M.; Zhang, H. Determinants of Severe Metabolic Bone Disease in Very Low-Birth-Weight Infants with Severe Bronchopulmonary Dysplasia Admitted to a Tertiary Referral Center. *Am. J. Perinatol*. **2016**, *33*, 107-113. https://doi.10.1055/s-0035-1560043.
213. Abend, N.S.; Monk, H.M.; Licht, D.J.; Dlugos, D.J. Intravenous levetiracetam in critically ill children with status epilepticus or acute repetitive seizures. *Pediatr. Crit. Care Med.* **2009**, *10*, 505-510. https://doi.10.1097/PCC.0b013e3181a0e1cf.
214. George, S.; Weber, D.R.; Kaplan, P.; Hummel, K.; Monk, H.M.; Levine, M.A. Short-Term Safety of Zoledronic Acid in Young Patients With Bone Disorders: An Extensive Institutional Experience. *J. Clin. Endocrinol. Metab*. **2015**, *100*, 4163-4171. https://doi.10.1210/jc.2015-2680.
215. Bamat, N.A.; Kirpalani, H.; Feudtner, C.; Jensen, E.A.; Laughon, M.M.; Zhang, H.; Monk, H.M.; Passarella, M.; Lorch, S.A. Medication use in infants with severe bronchopulmonary dysplasia admitted to United States children's hospitals. *J. Perinatol*. **2019**, *39*, 1291-1299. https://doi.10.1038/s41372-019-0415-9.
216. Monk, H.M.; Motsney, A.J.; Wade, K.C. Safety of rotavirus vaccine in the NICU. *Pediatrics.* **2014**, *133*, e1555-1560. https://doi.10.1542/peds.2013-3504.
162. Ravichandran, B.R.; Gillespie, M.W.; Sparkes, T.M.; Williams, C.; Bartlett, S.T.; Haririan, A.; Masters, B.M. Collaborative practice agreement in solid organ transplantation. Int. J. Clin. Pharm. 2018, 40, 474-479. https://doi.10.1007/s11096-018-0604-1.
217. Sparkes, T.; Lemonovich, T.; AST Infectious Diseases Community of Practice. Interactions between anti-infective agents and immunosuppressants-Guidelines from the American Society of Transplantation Infectious Diseases Community of Practice. *Clin. Transplant.* **2019**, *33*, e13510. https://doi.10.1111/ctr.13510.
218. Campara, M.; Lourenco, L.M.; Melaragno, J.I.; Kaiser, T.E. Implications for body weight extremes in solid organ transplantation. *Pharmacotherapy.* **2021**, *41*, 44-58. https://doi.10.1002/phar.2493.
219. Yakubu, I.; Ravichandran, B.; Sparkes, T.; Barth, R.N.; Haririan, A.; Masters, B. Comparison of Alemtuzumab Versus Basiliximab Induction Therapy in Elderly Kidney Transplant Recipients: A Single-Center Experience. *J. Pharm. Pract.* **2021**, *34*, 199-206. https://doi.10.1177/0897190019850934.
220. Hollis, I.B.; Reed, B.N.; Moranville, M.P. Medication management of cardiac allograft vasculopathy after heart transplantation. *Pharmacotherapy.* **2015**, *35*, 489-501. doi:10.1002/phar.1580.
221. Doligalski, C.T.; Anger, L.B.; Dick, T.; Feist, A.; Harris, M.; Masters, B.; Pilch, N.; Quan, D.; Sparkes, T.; Suarez, T.; Yakubu, I. Considerations and approaches to expansion of solid organ transplant pharmacist services. *J. Am. Coll. Clin. Pharm.* **2021**, *4*, 1445-1456. https://doi.10.1002/jac5.1514.
222. Ravichandran, B.R.; Sparkes, T.M.; Masters, B.M.; Thomas, B.; Demehin, M.; Bromberg, J.S.; Haririan, A. Survival benefit of renal transplantation in octogenarians. *Clin. Transplant.* **2020**, *34*, e14074. https://doi.10.1111/ctr.14074.
